# Atlas of Clinical Medicine

**Published:** 1892-06

**Authors:** 


*Atlas of Clinical Medicine.* By BYROM BRAMWELL, M.D., F.R.C.P., and F.R.S.E. Vol. I. Part III. Pp. 43. Edinburgh: T. AND A. CONSTABLE. 1891.

The subjects dealt with in this part are Progressive Unilateral Atrophy of the Face, Chronic Progressive Bulbar Paralysis, Ophthalmoplegia, Molluscum Fibrosum, and Xeroderma Pigmentosum. The plates of cases illustrating these affections are excellent, and maintain the high standard of the preceding numbers. The letterpress is also good. We think, however, that the account of ophthalmoplegia might be condensed, and that it should be more clearly pointed out what are the usual combinations of paralysis that are seen in practice as distinguished from those that may theoretically occur. On page 125, "splenoidal" is evidently a misprint for "sphenoidal."

